# A Retrospective Study of the Efficacy and Safety of Chemoimmunotherapy in Elderly Patients with Extensive-Stage Small Cell Lung Cancer

**DOI:** 10.3390/cancers18193131

**Published:** 2026-09-27

**Authors:** Minoru Kobayashi, Satoshi Watanabe, Shinji Hirose, Hiroki Watanabe, Kohei Kushiro, Ryo Suzuki, Naohiro Yanagimura, Miyuki Sato, Tomohiro Tanaka, Koichiro Nozaki, Yu Saida, Jun Arinami, Yuka Goto, Ko Sato, Daisuke Ishikawa, Takao Miyabayashi, Kosuke Ichikawa, Naoya Matsumoto, Jun Koshio, Yoshiki Hayashi, Kenichi Koyama, Masato Makino, Hideyuki Kuriyama, Ryohei Terashima, Atsushi Hashimoto, Toshiaki Kikuchi

**Affiliations:** 1 Department of Respiratory Medicine and Infectious Diseases, Niigata University Graduate School of Medical and Dental Sciences, 1-757 Asahimachidori, Chuouku, Niigata 951-8510, Japan; kobami733@yahoo.co.jp (M.K.); kokuama1230@gmail.com (S.H.); s.h48694062.base@gmail.com (H.W.); kuusan134@gmail.com (K.K.); ryo.suzuki.kij@niigata-u.ac.jp (R.S.); nyanagimura@med.niigata-u.ac.jp (N.Y.); miyuw0527@gmail.com (M.S.); tomtanak@med.niigata-u.ac.jp (T.T.); knozaki@med.niigata-u.ac.jp (K.N.); saida@med.niigata-u.ac.jp (Y.S.); kikuchi@med.niigata-u.ac.jp (T.K.); 2 Nagaoka Red Cross Hospital, Nagaoka 940-2085, Japan; jyaumkairi5463@gmail.com (J.A.); jun_koshio@yahoo.co.jp (J.K.); 3 Niigata Prefectural Shibata Hospital, Shibata 957-8588, Japan; yuka1009g@gmail.com (Y.G.); sevenseavoyage@outlook.jp (M.M.); 4 Joetsu General Hospital, Joetsu 943-8507, Japan; m03a037c@yahoo.co.jp; 5 Niigata Prefectural Central Hospital, Joetsu 943-019, Japan; lapistecclazuli@gmail.com; 6 Niigata City General Hospital, Niigata 950-1197, Japan; tmiyaba5538@yahoo.co.jp; 7 Saiseikai Niigata Hospital, Niigata 950-1104, Japan; wrx5772@yahoo.co.jp; 8 Nishiniigata Chuo Hospital, Niigata 950-2085, Japan; matsumoto.naoya.dx@mail.hosp.go.jp; 9 Nagaoka Chuo General Hospital, Nagaoka 940-0861, Japan; yyhayashiyy@yahoo.co.jp; 10 Niigata Cancer Center Hospital, Niigata 951-8133, Japan; k-koyama@niigata-cc.jp; 11 Niigata Medical Center, Niigata 950-2022, Japan; actliberation46@yahoo.co.jp; 12 Clinical Research Center, Niigata University Medical and Dental Hospital, 1-754 Asahimachidori, Chuouku, Niigata 951-8520, Japan; ryouheitera.wv7@nuh.niigata-u.ac.jp (R.T.); ashlivex@med.niigata-u.ac.jp (A.H.)

**Keywords:** extensive-stage small cell lung cancer, PD-L1 inhibitor, elderly patients

## Abstract

**Background/Objectives:** We evaluated the efficacy and safety of first-line treatment with a programmed death-ligand 1 (PD-L1) inhibitor plus platinum-based chemotherapy versus chemotherapy alone in patients aged ≥75 years with extensive-stage small cell lung cancer (ES-SCLC). **Methods:** We retrospectively analyzed the patients with ES-SCLC treated within the Niigata Lung Cancer Treatment Study Group between August 2019 and April 2024. **Results:** A total of 364 consecutive patients were enrolled. After excluding 21 patients who did not receive treatment or had ineligible histological types, 343 patients were included in the analysis. Of these, 132 were aged ≥75. Compared with chemotherapy alone, administering a PD-L1 inhibitor with chemotherapy significantly improved the median overall survival among elderly patients (13.6 vs. 8.4 months; hazard ratio, 0.543; *p* = 0.0277). The median progression-free and overall survivals were similar between the ≥75-year and <75-year groups treated with PD-L1 inhibitor plus chemotherapy (4.9 vs. 4.8 months; *p* = 0.7611 and 13.6 vs. 15 months; *p* = 0.732, respectively). Immune-related adverse events (irAE) of ≥grade 3 occurred in 4 and 12 patients (*p* = 0.7823), and non irAE of ≥grade 3 occurred in 47 and 103 patients (*p* = 0.5247) in the ≥75-years and <75-years subgroups, respectively. **Conclusions:** In this retrospective study, among patients aged ≥75 years with ES-SCLC, the addition of a PD-L1 inhibitor to platinum-based chemotherapy was associated with longer overall survival. Progression-free and overall survival outcomes were comparable between elderly and younger patients, with no clear age-related difference observed in the incidence of adverse events of ≥grade 3.

## Simple Summary

This retrospective study evaluated the effectiveness and safety of adding a programmed death-ligand 1 (PD-L1) inhibitor to platinum-based chemotherapy in patients aged ≥75 year with extensive-stage small cell lung cancer. The combination treatment significantly prolonged overall survival compared with chemotherapy alone. The incidence of severe adverse events was comparable between patients aged ≥75 years and younger patients. These findings suggest that adding a PD-L1 inhibitor to chemotherapy appears to be an effective and tolerable first-line treatment option for elderly patients with extensive-stage small cell lung cancer.

## 1. Introduction

Regimens combining cisplatin (CDDP) or carboplatin (CBDCA) with etoposide (ETP), as well as those combining CDDP or CBDCA with irinotecan, have long been recommended as first-line treatments for extensive-stage small-cell lung cancer (ES-SCLC). For nearly two decades, no systemic therapy had demonstrated superiority over these regimens. However, the IMpower133 and CASPIAN trials showed that the addition of a programmed death-ligand 1 (PD-L1) inhibitor to platinum–ETP chemotherapy significantly improved overall survival (OS) and progression-free survival (PFS). Consequently, PD-L1 inhibitor-based chemoimmunotherapy has become a standard first-line treatment for patients with untreated ES-SCLC [1,2].

The safety and efficacy of treatment in elderly patients may differ from those in younger patients because elderly individuals more frequently have comorbidities and reduced organ function. Although the IMpower133 and CASPIAN trials did not impose an upper age limit, the median ages of enrolled patients were 64 and 62 years, respectively. In IMpower133, patients aged ≥75 years accounted for approximately 10% of the study population. In contrast, a retrospective real-world study of patients with ES-SCLC in Japan reported a median age of 72 years, with patients aged ≥75 years comprising 31% of the cohort. This finding indicates that the Japanese real-world cohort included a substantially higher proportion of elderly patients than the populations enrolled in the IMpower133 and CASPIAN trials [3].

In the randomized phase III OAK trial of non-small-cell lung cancer (NSCLC), no clinically meaningful age-related difference in the safety of immune checkpoint inhibitor (ICI) monotherapy was observed: grade 3–4 treatment-related adverse events (AEs) occurred in 14.1% of patients aged <65 years and 15.6% of those aged ≥65 years [4]. Similarly, a pooled analysis of the KEYNOTE-010, KEYNOTE-024, and KEYNOTE-042 trials showed no increase in AEs among patients aged ≥75 years and no attenuation of the OS benefit associated with pembrolizumab monotherapy in this population [5]. In contrast, the efficacy of chemoimmunotherapy for NSCLC may be reduced in elderly patients. In KEYNOTE-189, which evaluated the addition of pembrolizumab to platinum-based chemotherapy, the hazard ratio (HR) for OS was 0.42 in patients aged <65 years and 0.63 in those aged ≥65 years, suggesting a smaller relative benefit in the elderly subgroup [6]. Furthermore, in a retrospective study of advanced NSCLC, CBDCA plus pemetrexed and pembrolizumab was associated with significantly shorter OS and PFS in patients aged ≥75 years than in those aged <75 years [7].

In ES-SCLC, a meta-analysis of six phase III trials, including CASPIAN and IMpower133, comparing programmed cell death 1 (PD-1)/PD-L1 inhibitor-based combination therapy with platinum-based chemotherapy demonstrated a significant OS benefit in patients aged ≥65 years (HR, 0.80; 95% confidence interval [CI], 0.72–0.90) [8]. In Japan, the prospective real-world J-TAIL-2 study evaluated atezolizumab plus CBDCA and ETP in a cohort that included a high proportion of elderly patients aged ≥70 years and patients who did not meet the eligibility criteria of IMpower133 (72.7%). In this clinically heterogeneous population, the median OS was 16.5 months (95% CI, 14.9–18.2). The median OS was 16.4 months in patients aged ≥70 years and 17.9 months in those aged <70 years, with no statistically significant difference between the groups (HR, 1.18; 95% CI, 0.90–1.55) [9]. However, because this was a single-arm observational study, it did not permit a direct comparison of the efficacy and safety of chemotherapy alone versus PD-L1 inhibitor-based chemoimmunotherapy.

Nevertheless, evidence regarding the efficacy and safety of chemoimmunotherapy in elderly patients aged ≥75 years with ES-SCLC remains limited, particularly because this subgroup was underrepresented in previous phase III trials. Establishing the optimal first-line treatment for this population therefore remains an important unmet clinical need [10]. In addition, several studies in NSCLC have suggested that the benefit of chemoimmunotherapy may be smaller in elderly patients than in younger patients. Therefore, in this retrospective study, we compared the efficacy of PD-L1 inhibitor-based chemoimmunotherapy with that of chemotherapy alone in elderly patients aged ≥75 years with ES-SCLC. We also compared the efficacy and safety of PD-L1 inhibitor-based chemoimmunotherapy between elderly and younger patients with ES-SCLC.

## 2. Materials and Methods

### 2.1. Study Design

This retrospective multicenter cohort study was conducted at institutions participating in the Niigata Lung Cancer Treatment Group. We included patients treated at participating institutions between 22 August 2019, and 30 April 2024, who met the predefined inclusion criteria and none of the exclusion criteria. Eligible patients had extensive-stage small-cell lung cancer (ES-SCLC) confirmed histologically or cytologically. Patients with mixed histology who were treated according to an ES-SCLC treatment strategy were also eligible. Data on patients with ES-SCLC who received best supportive care alone without systemic anticancer therapy were also collected during case report form collection. However, these patients were excluded from the final analysis because the study population was restricted to patients who received systemic treatment. Patients were excluded from the final analysis if they had ineligible histological types, limited-stage SCLC treated with surgery, definitive radiotherapy, or chemotherapy, or had participated in interventional clinical trials. Participation in observational studies was permitted. We collected data on age, sex, smoking history, performance status (PS), presence and sites of metastases, histological subtype, interstitial lung disease or interstitial lung abnormalities on imaging, autoimmune disease, number of drug administrations, date of final drug administration, number of treatment cycles, treatment history, occurrence of grade ≥3 adverse events (AEs), occurrence of grade ≥3 immune-related adverse events (irAEs), concomitant use of proton pump inhibitors, potassium-competitive acid blockers, antibiotics, probiotics, and antihistamines, final outcome, date of last follow-up or death, and cause of death. Patients aged ≥75 years were defined as elderly, and a PS of 2–4 was defined as poor PS. Standard doses were defined as CDDP at 80 mg/m^2^, CBDCA at an area under the curve (AUC) of 5, and ETP at 100 mg/m^2^. The primary endpoint was the difference in real-world overall survival (rwOS) between elderly patients with ES-SCLC who received a PD-L1 inhibitor plus platinum-based chemotherapy and those who received chemotherapy alone. Secondary endpoints were real-world progression-free survival (rwPFS); safety, including grade ≥ 3 AEs, grade ≥ 3 irAEs, and treatment-related mortality; the number of administrations of PD-L1 inhibitor maintenance therapy; time to treatment failure; the association between irAEs and treatment efficacy; and the association of concomitant oral medications—including proton pump inhibitors, potassium-competitive acid blockers, antibiotics, probiotics, and antihistamines—with treatment efficacy.

This study was conducted in accordance with the Declaration of Helsinki, as revised in October 2013. The study protocol was approved by the Niigata University Clinical Research Ethics Committee (approval no. 2024-0242; 29 January 2025) and was registered with the University Hospital Medical Information Network (UMIN; registration no. UMIN000060603). The requirement for individual informed consent was waived because of the retrospective nature of the study. Information regarding the study was disclosed through an opt-out procedure, and patients were provided with an opportunity to decline participation.

### 2.2. Study Assessment

The observation period was defined as the period from treatment initiation to the data cutoff date (30 April 2025). Radiologic assessments were generally performed after every two cycles; however, the timing of these assessments was determined at the discretion of the treating physician. Tumor responses were also assessed by treating physicians according to the Response Evaluation Criteria in Solid Tumors, version 1.1. PFS was defined as the time from treatment initiation until disease progression or death from any cause. OS was defined as the time from treatment initiation until death from any cause. Patients who were alive without disease progression at the last follow-up were censored for the PFS analysis, whereas patients who were alive at the last follow-up were censored for the OS analysis. AEs, including irAEs, were graded according to the National Cancer Institute Common Terminology Criteria for Adverse Events, version 5.0.

### 2.3. Statistical Analysis

PFS and OS were estimated using the Kaplan–Meier method and compared between groups using the log-rank test. Hazard ratios (HRs) and their 95% confidence intervals were estimated using Cox proportional hazards models. Prognostic factors were assessed using univariate and multivariable Cox proportional hazards regression analyses. All statistical tests were two-sided, and a *p* value of <0.05 was considered statistically significant. All analyses were performed using JMP^®^ Pro version 18.2.2 (SAS Institute Inc., Cary, NC, USA).

## 3. Results

### 3.1. Participants

A total of 364 consecutive patients were included in the case report form collection. After the exclusion of 21 patients because of ineligible histological types or receipt of best supportive care alone without systemic anticancer therapy, 343 patients were included in the final analysis. During the data collecting period from 2019 to 2024, approximately 50% of patients aged ≥75 years received chemoimmunotherapy in each year. Among patients aged <75 years, approximately 50–60% received chemoimmunotherapy in 2019 and 2020, with this proportion increasing to approximately 70–80% from 2021 onward (Supplementary Table S1). The median follow-up was 27.5 months (95% CI, 20.5–30.6), estimated using reverse Kaplan–Meier method. Of these, 132 patients (39%) were aged ≥75 years. Among patients aged ≥75 years, 65 received PD-L1 inhibitor-based chemoimmunotherapy, whereas 67 received platinum-based chemotherapy alone (Figure 1). Approximately 85% of patients in both the ≥75 and <75 years groups were male, and most had a history of smoking (Table 1). ILD was present in 24% of patients aged ≥75 years, which was significantly higher than the proportion among patients aged <75 years (13%; *p* = 0.0080). No significant differences were observed between the age groups in the prevalence of brain or liver metastases. The proportion of patients who received PD-L1 inhibitor-based chemoimmunotherapy was significantly lower in the elderly group than in the younger group (50% vs. 73%, respectively; *p* < 0.0001). Reasons for not receiving PD-L1 inhibitors are presented in Supplementary Table S2. The most common reason for not adding a PD-L1 inhibitor was the presence of ILD in both patients aged ≥75 years (37%) and those aged <75 years (36%). Other common reasons in both age groups included poor PS and organ dysfunction.

### 3.2. Efficacy

In the overall cohort, PFS was significantly longer in the PD-L1 inhibitor-based chemoimmunotherapy group than in the chemotherapy-alone group (HR, 0.7144; 95% CI, 0.5670–0.9001; *p* = 0.0043; Figure 2A). Similarly, OS was significantly longer in the PD-L1 inhibitor-based chemoimmunotherapy group than in the chemotherapy-alone group (HR, 0.5863; 95% CI, 0.4513–0.7618; *p* < 0.0001; Figure 2B). When stratified according to age (<75 vs. ≥75 years) and treatment group, the PFS and OS curves for the PD-L1 inhibitor-based chemoimmunotherapy groups were consistently above those for the chemotherapy-alone groups, with a more pronounced separation observed for OS (Figure 2C,D).

Chemo, chemotherapy; CI, confidence interval; HR, hazard ratio; ICI, immune checkpoint inhibitor; OS, overall survival; PD-L1, programmed death-ligand 1; PFS, progression free survival.

Among patients who received PD-L1 inhibitor-based chemoimmunotherapy, no significant differences in PFS or OS were observed between patients aged ≥75 years and those aged <75 years (Supplementary Figure S1A,B). Among patients aged ≥75 years, no patients in the PD-L1 inhibitor-based chemoimmunotherapy group had ILD, whereas ILD was present in 48% of patients in the chemotherapy-alone group (Supplementary Table S3). Because ILD was unevenly distributed between the treatment groups, we performed an analysis restricted to patients aged ≥75 years without ILD. In this analysis, the median PFS was 4.9 months (95% CI, 4.2–5.6) in the PD-L1 inhibitor-based chemoimmunotherapy group and 4.6 months (95% CI, 3.3–5.8) in the chemotherapy-alone group (HR, 0.7933; 95% CI, 0.5108–1.2139; *p* = 0.3024; Figure 3A). The median OS was 13.6 months (95% CI, 10.9–19.3) and 8.4 months (95% CI, 4.6–12.8), respectively (HR, 0.5430; 95% CI, 0.3153–0.9351; *p* = 0.0277; Figure 3B).

In the analysis including patients with ILD, the median PFS was 4.9 months (95% CI, 4.2–5.6) in the PD-L1 inhibitor-based chemoimmunotherapy group and 4.3 months (95% CI, 3.2–5.3) in the chemotherapy-alone group (HR, 0.7443; 95% CI, 0.5167–1.0720; *p* = 0.1100; Supplementary Figure S2A). The corresponding median OS was 13.6 months (95% CI, 10.9–19.3) and 8.9 months (95% CI, 5.7–11.7), respectively (HR, 0.5726; 95% CI, 0.3696–0.8870; *p* = 0.0125; Supplementary Figure S2B). Thus, the analysis including patients with ILD showed results consistent with those of the analysis restricted to patients without ILD: OS was significantly longer in the PD-L1 inhibitor-based chemoimmunotherapy group, whereas the difference in PFS was not statistically significant.

Univariable Cox proportional hazards analyses were performed to evaluate the associations between OS and baseline factors previously reported to be associated with ICI efficacy in patients with advanced NSCLC (Table 2). Poor PS and the presence of liver metastases were significantly associated with shorter OS in univariable analyses. In multivariable analysis including these two factors, both poor PS and liver metastases remained independently associated with shorter OS. In addition, we performed univariable and multivariable analyses of OS among patients aged ≥75 years, including the use of a PD-L1 inhibitor as a covariate (Supplementary Table S4). The addition of a PD-L1 inhibitor and poor PS were independently associated with OS.

Because poor PS was consistently associated with shorter OS in both the univariable and multivariable analyses, we further evaluated whether PS modified the association between PD-L1 inhibitor-based chemoimmunotherapy and survival outcomes in patients aged ≥75 years with ES-SCLC. Among patients with good PS, PD-L1 inhibitor-based chemoimmunotherapy was associated with significantly longer OS, whereas the difference in PFS was not statistically significant (Supplementary Figure S3A,B). In contrast, among patients with poor PS, no statistically significant differences in PFS or OS were observed between the treatment groups (Supplementary Figure S3C,D).

### 3.3. Safety

Only grade ≥3 AEs were collected in this study (Table 3). Among patients who received PD-L1 inhibitor-based chemoimmunotherapy, the incidence of grade ≥ 3 irAEs did not differ significantly between patients aged ≥75 years and those aged <75 years (*n* = 4 (6%) vs. n = 12 (8%), respectively; *p* = 0.7823). Similarly, the incidence of grade ≥ 3 non-irAEs was not significantly different between the ≥75 and <75 years groups (*n* = 48 (74%) vs. n = 103 (67%), respectively; *p* = 0.4227). Data on G-CSF use were collected; however, information on whether G-CSF was administered as primary or secondary prophylaxis was unavailable. G-CSF was administered to 55% of patients aged 75 years and 45% of those aged 75 years, with no significant difference between the groups (*p* = 0.7713). In contrast, chemotherapy dose reductions were significantly more frequent among patients aged ≥75 years (75% vs. 30%, *p* < 0.0001). Notably, consistent with previous findings in NSCLC, patients with SCLC who developed grade ≥ 3 irAEs during chemoimmunotherapy had significantly longer OS (24.4 vs. 14.3 months, respectively; *p* = 0.0484).

We compared completion of four cycles, transition to maintenance therapy, and early death before completion of induction therapy between patients aged ≥75 years and <75 years. The proportion of patients who completed four cycles was significantly lower in the older group (66% vs. 83%, *p* = 0.006), whereas the proportion who transitioned to maintenance therapy did not differ significantly between the groups (62% vs. 73%, *p* = 0.11). Early death before completion of induction therapy was significantly more frequent in patients aged ≥75 years (18% vs. 9%, *p* = 0.0347).

We further compared AEs between chemoimmunotherapy and chemotherapy alone among patients aged ≥75 years (Supplementary Table S5). No significant differences were observed between the chemoimmunotherapy and chemotherapy-alone groups in the incidence of individual AEs, including neutropenia, febrile neutropenia and ILD. Although grade ≥3 AEs were significantly more frequent in the chemoimmunotherapy group than in the chemotherapy-alone group (37% vs. 18%, *p* = 0.0188), the incidence of AEs leading to discontinuation of first-line treatment or death did not differ significantly between the two groups.

### 3.4. Subsequent Treatments

Elderly patients often have impaired organ function and multiple comorbidities and may experience a rapid decline in their general condition because of disease progression or treatment-related complications. This may reduce their likelihood of receiving subsequent therapy. We therefore evaluated treatment patterns and the proportions of patients who received subsequent lines of therapy. Among patients who received PD-L1 inhibitor-based chemoimmunotherapy as first-line treatment, 58% of patients aged ≥75 years and 71% of those aged <75 years received second-line or later therapy (Supplementary Table S6; *p* = 0.0861). The proportions of patients who received third-line therapy were 23% and 46%, respectively (*p* = 0.0022), whereas those who received fourth-line therapy were 3% and 23%, respectively (*p* = 0.0001). Thus, among patients who received first-line PD-L1 inhibitor-based chemoimmunotherapy, elderly patients were significantly less likely than younger patients to receive third-line or fourth-line therapy. Similarly, among patients who received chemotherapy alone as first-line treatment, the proportions of patients who received subsequent lines of therapy were significantly lower in those aged ≥75 years than in those aged <75 years (Supplementary Table S6).

## 4. Discussion

Previous phase III trials and real-world studies have demonstrated that the addition of a PD-L1 inhibitor to chemotherapy improves OS in the overall population with ES-SCLC [1,2]. However, evidence regarding the efficacy and safety of this regimen in patients aged ≥75 years, particularly evidence from direct comparisons with chemotherapy alone, remains limited. With progressive population aging, the number of patients aged ≥75 years with ES-SCLC is expected to increase. In the present study, patients aged ≥75 years accounted for 38% of the overall cohort, which was comparable to the proportion of 31% reported in a previous Japanese real-world study [3]. To our knowledge, this study is one of the largest retrospective real-world analyses comparing PD-L1 inhibitor-based chemoimmunotherapy with chemotherapy alone in patients aged ≥75 years with ES-SCLC. In subgroup analyses of the CASPIAN trial, the HR for OS with durvalumab plus chemotherapy versus chemotherapy alone was 0.71 (95% CI, 0.58–0.88) among patients aged <70 years and 0.74 (95% CI, 0.49–1.11) among those aged ≥70 years, suggesting a numerically favorable association in the elderly subgroup, although the CI was wide [11]. Similarly, in IMpower133, the HRs for OS were 0.86 (95% CI, 0.62–1.18) among patients aged <65 years and 0.53 (95% CI, 0.36–0.77) among those aged ≥65 years. Although these subgroup analyses should be interpreted cautiously, they do not suggest an attenuation of the survival benefit of PD-L1 inhibitor-based chemoimmunotherapy with increasing age [1]. In advanced NSCLC, the efficacy of ICIs in elderly patients may vary according to the treatment strategy, including ICI monotherapy, chemoimmunotherapy, and regimens containing cytotoxic T-lymphocyte-associated protein 4 (CTLA-4) inhibitors. A pooled analysis of the KEYNOTE-010, KEYNOTE-024, and KEYNOTE-042 trials, which evaluated pembrolizumab monotherapy in patients with PD-L1-positive advanced NSCLC, showed comparable efficacy in patients aged ≥75 years and those aged <75 years [5]. By contrast, the NEJ057 study, which compared ICI monotherapy, ICI plus chemotherapy, and chemotherapy alone in elderly patients with NSCLC, showed similar outcomes in the ICI monotherapy and ICI-plus-chemotherapy groups [12]. In particular, among patients with a PD-L1 tumor proportion score (TPS) of 1–49% or ≥50%, the addition of chemotherapy did not provide a further OS benefit, suggesting that ICI monotherapy may be an appropriate option for selected elderly patients. In the CheckMate 227 and CheckMate 9LA trials, which evaluated nivolumab plus ipilimumab with or without platinum-doublet chemotherapy in advanced NSCLC, the HRs for OS among patients aged ≥75 years were 0.92 (95% CI, 0.57–1.48) and 1.21 (95% CI, 0.69–2.12), respectively [13,14]. Taken together, these findings suggest that the benefit–risk balance of combination therapy may be less favorable in some elderly patients, particularly those with limited treatment tolerance.

Previous real-world studies have yielded inconsistent findings regarding the benefit of adding ICIs in older patients with ES-SCLC. Zhao et al. reported that, among patients aged ≥70 years, chemoimmunotherapy significantly prolonged PFS compared with chemotherapy alone, whereas the improvement in OS did not reach statistical significance. [15] Similarly, Dong et al. observed numerically longer PFS and OS with chemoimmunotherapy among patients aged ≥75 years, although neither difference was statistically significant. [16] In this study, PD-L1 inhibitor-based chemoimmunotherapy was associated with significantly longer OS than chemotherapy alone among patients aged ≥75 years with ES-SCLC. Among elderly patients who received PD-L1 inhibitor-based chemoimmunotherapy, the median PFS and OS were 4.9 and 13.6 months, respectively. Although the difference in PFS between the PD-L1 inhibitor-based chemoimmunotherapy and chemotherapy-alone groups was not statistically significant in this elderly cohort, OS was significantly longer in the PD-L1 inhibitor-based chemoimmunotherapy group. These outcomes were comparable to those reported in the overall populations of IMpower133, which evaluated atezolizumab plus platinum–ETP chemotherapy (median PFS, 5.2 months; median OS, 12.3 months), and CASPIAN, which evaluated durvalumab plus platinum–ETP chemotherapy (median PFS, 5.1 months; median OS, 13.0 months) [1,2]. Whereas IMpower133 and some real-world studies reported significant PFS prolongation following the addition of a PD-L1 inhibitor, CASPIAN and other real-world studies demonstrated an OS benefit without a statistically significant difference in PFS [1,2,17,18]. This discordance between PFS and OS may indicate that PD-L1 inhibitors provide benefits beyond initial disease control, potentially through sustained immune modulation. The survival outcomes observed in our elderly cohort were comparable to those reported in pivotal phase III trials involving unselected populations. These findings suggest that chronological age alone may not preclude benefit from PD-L1 inhibitor-based chemoimmunotherapy in patients with ES-SCLC. In contrast, the additional benefit associated with PD-L1 inhibitor-based chemoimmunotherapy appeared to be limited among patients aged ≥75 years with poor PS. Our research group previously reported that PD-L1 inhibitor-based chemoimmunotherapy could be administered safely and effectively to patients with ES-SCLC and PS 2–3 through adjustment of chemotherapy doses [19]. However, in that study, the incremental benefit of PD-L1 inhibitors was limited among patients whose PS deterioration was attributable to comorbidities. These findings suggest that treatment decisions for elderly patients should consider individual clinical characteristics, including PS and comorbidities, rather than age alone.

A higher proportion of patients aged ≥75 years in the present study had concomitant ILD, consistent with previous reports indicating that the incidence of ILD increases with age [20]. None of the patients with ILD received PD-L1 inhibitor-containing regimens as first-line therapy, suggesting that ILD substantially influenced treatment selection. In a cohort of patients with interstitial lung abnormalities, the incidence of treatment-related interstitial pneumonitis was significantly higher among patients who received PD-L1 inhibitor-based chemoimmunotherapy than among those without interstitial lung abnormalities [21]. Moreover, a Japanese multicenter retrospective study reported that 27% of patients with SCLC had concomitant ILD, and several studies have shown that ILD is associated with a poorer prognosis in this population. [22,23,24,25] Therefore, we performed analyses both in the overall cohort, including patients with ILD, and in the subgroup excluding patients with ILD to account for the potential influence of ILD on treatment selection and survival outcomes.

In IMpower133, grade ≥3 AEs occurred in 58% of patients who received atezolizumab plus chemotherapy, with neutropenia (23%), anemia (14%), and thrombocytopenia (10%) being the most common toxicities [1]. In CASPIAN, grade ≥ 3 AEs occurred in 67% of patients in the durvalumab plus chemotherapy group; neutropenia (24%), anemia (9%), and thrombocytopenia (6%) were the most frequently reported events [2]. Although the overall incidence of grade ≥ 3 AEs in the present study was higher than that reported in these trials, this difference may reflect the inclusion of a substantial proportion of patients with poor PS or organ dysfunction. Among elderly patients, 49 of 66 patients (74%) received an ETP dose reduction to 80 mg/m^2^ based on JCOG9702, and 15 patients underwent additional dose reductions of CBDCA or ETP [26]. These dose modifications may have contributed to the absence of a significant difference in the incidence of grade ≥3 neutropenia between elderly and younger patients. Notably, despite these dose reductions, survival outcomes in the elderly cohort were comparable to those reported in previous pivotal studies. The LOGiK2003 and NEJ045A trials, which enrolled elderly patients or those with poor PS and ES-SCLC, reported grade ≥ 3 AEs in 94.6% and 93.0% of patients, respectively, indicating that severe AEs are common in these clinically vulnerable populations. Neutropenia was the most frequent grade ≥ 3 AE in both trials, occurring in 76.3% and 61.0% of patients, respectively [19,27]. Appropriate supportive care and careful monitoring are therefore essential when treating elderly patients and patients with poor PS. In the present study, grade ≥ 3 irAEs occurred in 6% of patients aged ≥75 years and 8% of those aged <75 years. The incidence of grade ≥ 3 irAEs in pivotal trials of PD-L1 inhibitor-based chemoimmunotherapy for ES-SCLC was similarly low, at approximately 5%, and was broadly comparable to that observed in the present study [28]. In an observational study including patients with NSCLC, melanoma, and renal cell carcinoma, the incidence of irAEs did not differ between patients aged ≥70 years and those aged <70 years, with a numerically lower incidence observed in the elderly cohort [29]. Similarly, in the present real-world cohort, the incidence of grade ≥ 3 irAEs was comparable between elderly and younger patients with ES-SCLC. In NSCLC, the development of irAEs has been reported to be associated with prolonged OS [30]. A similar association between irAE development and improved OS has also been suggested in SCLC [31,32]. In the present study, among patients who received PD-L1 inhibitor-based chemoimmunotherapy, median OS was significantly longer in patients who developed grade ≥ 3 irAEs than in those without irAEs. This finding is consistent with previous reports. However, this result should be interpreted cautiously, as only 16 of the 218 patients developed grade ≥ 3 irAEs. In addition, because the development of irAEs requires patients to remain on treatment and alive for a certain period, time-dependent bias, including immortal time bias, cannot be excluded. Notably, 10 of the 16 patients who developed grade ≥ 3 irAEs did not receive second-line or subsequent therapy, yet an association between the development of grade ≥ 3 irAEs and prolonged OS was observed. The clinical implications of this association warrant further investigation in larger cohorts, ideally using analyses that account for the time-dependent nature of irAE occurrence.

This study has several limitations. First, its retrospective design makes it inherently susceptible to selection bias. Treatment selection, the assessment of ILD status, and AE evaluation were determined by the attending physicians, potentially resulting in residual confounding. Furthermore, the number of patients included in the safety analysis was relatively small in each group. Second, AE data collection was limited to grade ≥ 3 events. Therefore, lower-grade toxicities may have had unrecorded effects on activities of daily living and quality of life, particularly among elderly patients and those with poor PS. Third, the observed difference in OS between treatment groups may reflect not only treatment efficacy but also unmeasured factors that influenced treatment selection. For example, physicians may have selected patients for chemoimmunotherapy on the basis of clinical characteristics not captured in this retrospective analysis, including subtle differences in overall condition, comorbidity severity, or social support. Therefore, causal inferences cannot be drawn from these associations, and prospective validation or the accumulation of additional real-world evidence is warranted.

## 5. Conclusions

In conclusion, PD-L1 inhibitor-based chemoimmunotherapy was associated with improved OS compared with chemotherapy alone in patients aged ≥75 years with ES-SCLC. In addition, the efficacy and safety of PD-L1 inhibitor-based chemoimmunotherapy appeared broadly comparable between patients aged ≥75 years and those aged <75 years.

## Figures and Tables

**Figure 1:**
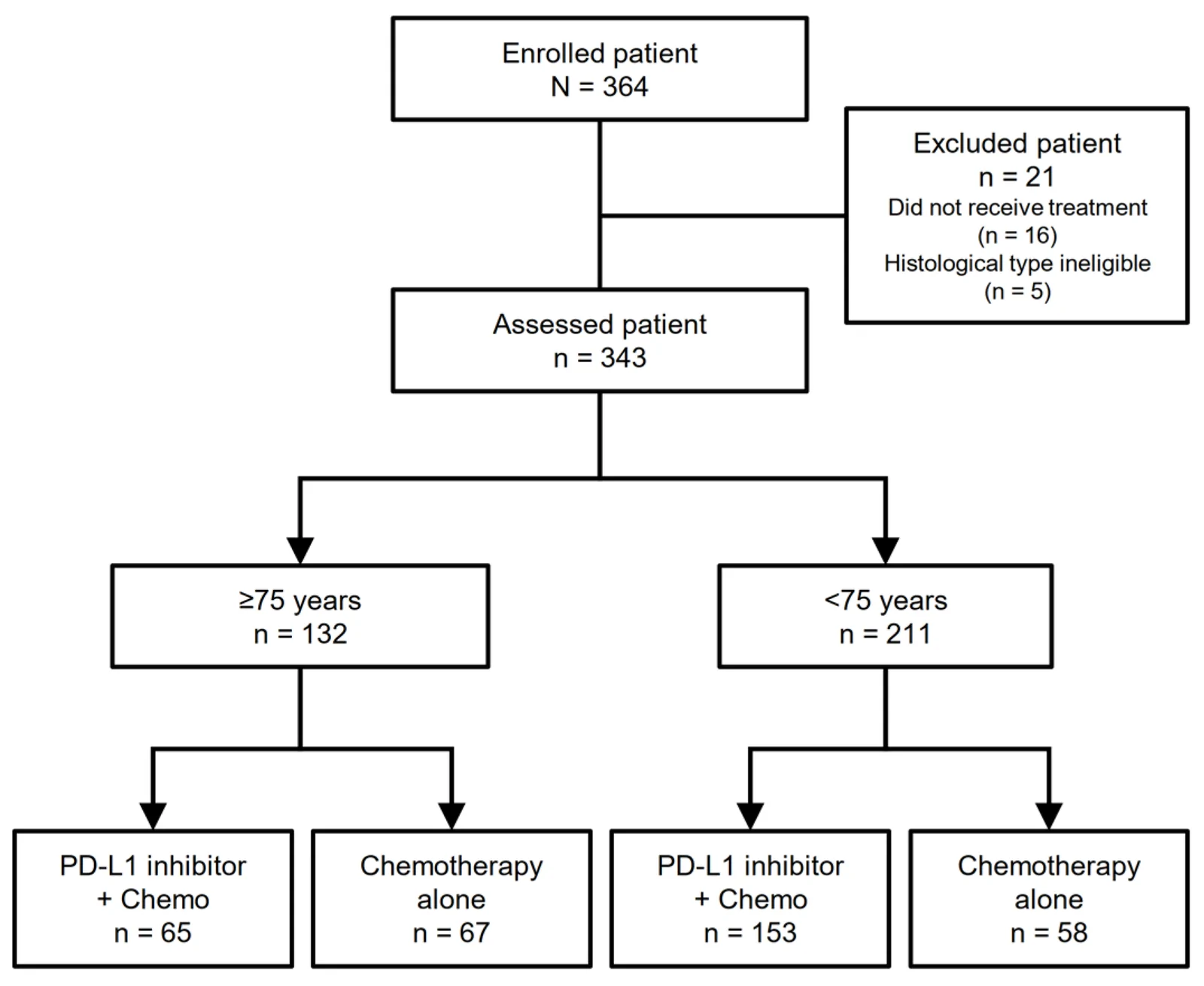
Flow diagram showing the selection of patients.

**Figure 2:**
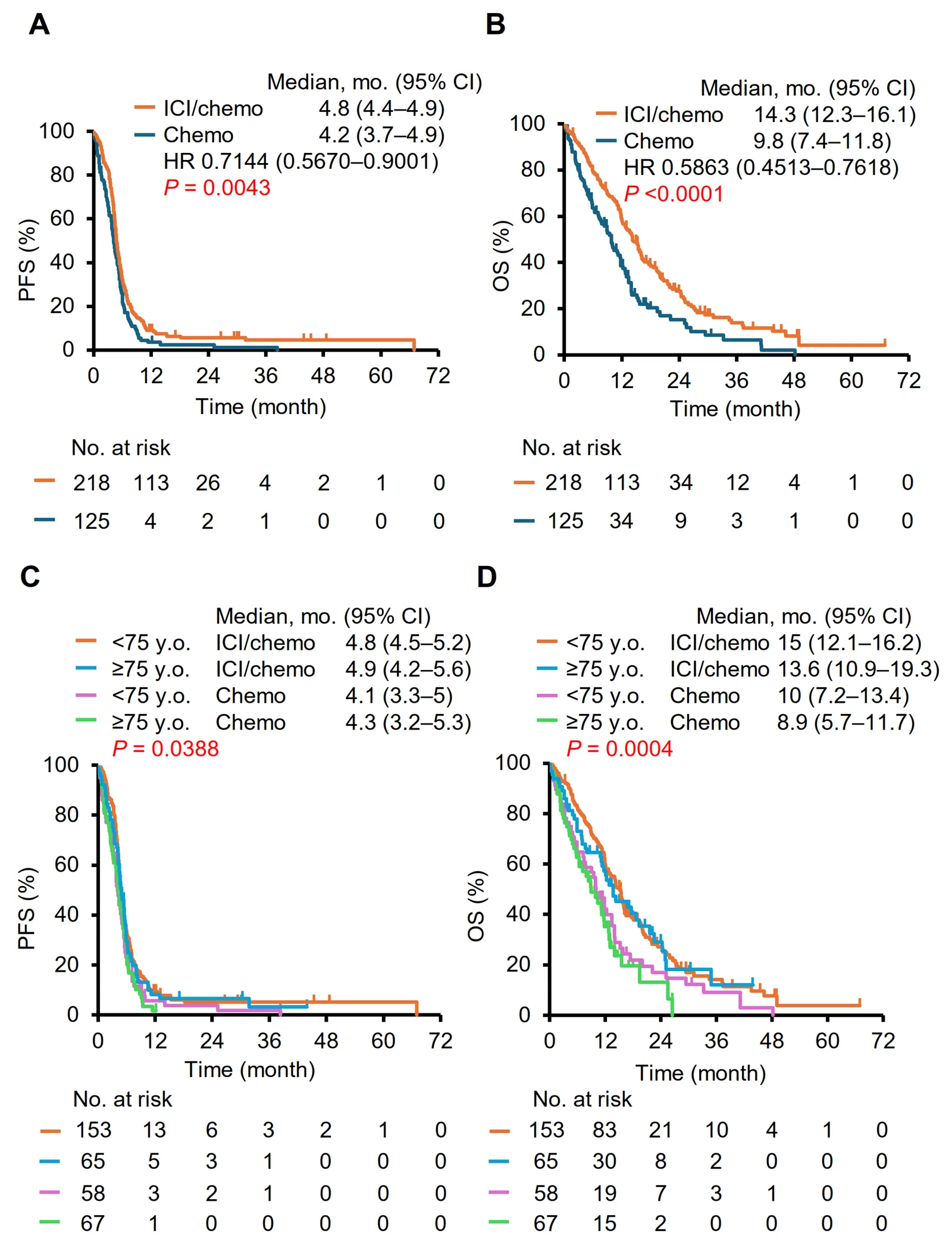
Outcomes in the overall population and in subpopulations stratified by age and treatment administered. Analyses of PFS (**A**) and OS (**B**) enrolled patients who were treated with a PD-L1 inhibitor plus chemotherapy or chemotherapy alone. Subgroup analyses of PFS (**C**) and OS (**D**) according to the four groups defined by age (≥75 vs. <75 years) and treatment (with vs. without concomitant administration of a PD-L1 inhibitor).

**Figure 3:**
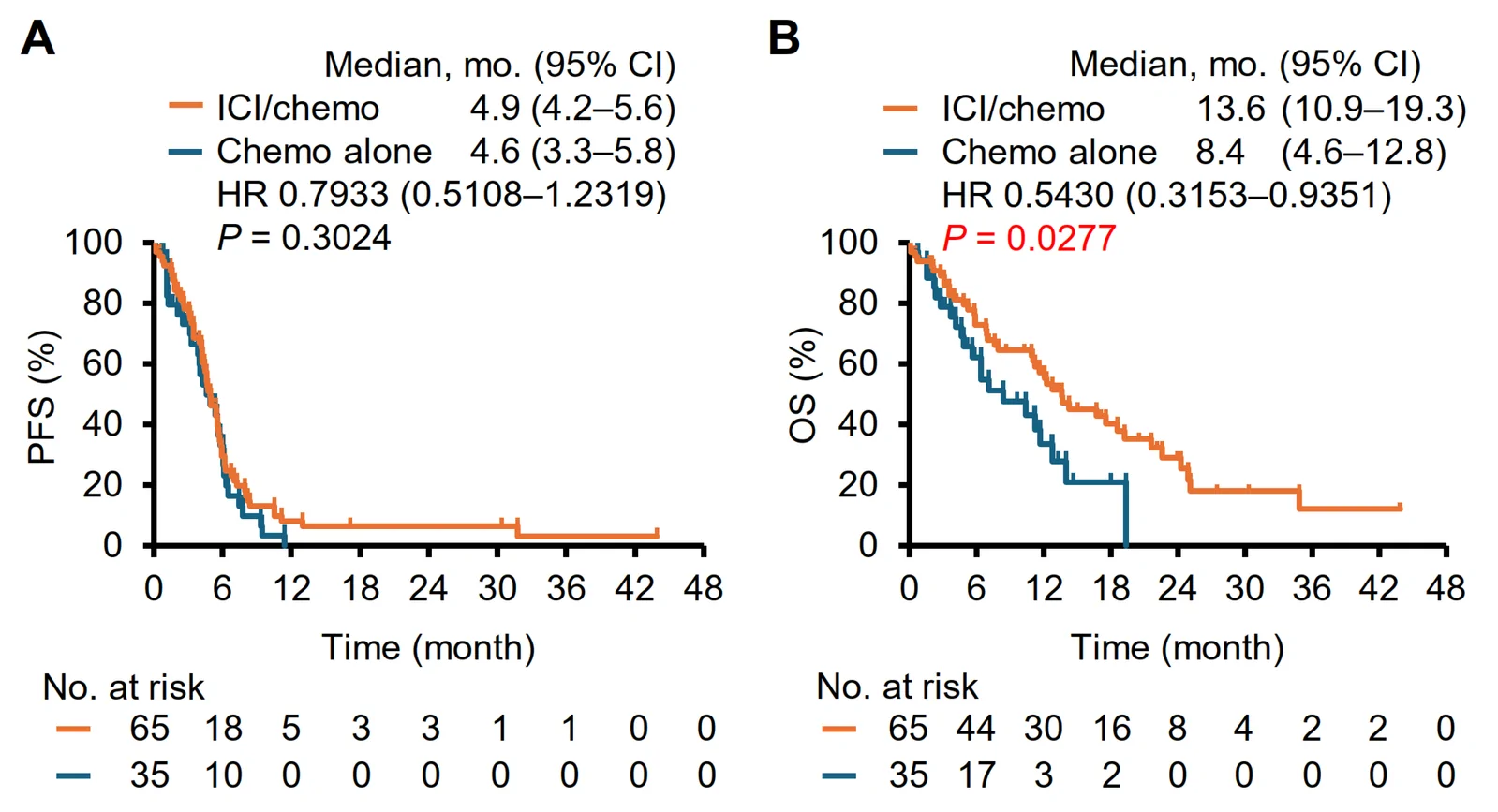
Outcomes in the population of patients aged ≥75 treated with a PD-L1 inhibitor plus chemotherapy or chemotherapy alone after excluding those with interstitial lung disease. Subgroup analyses of PFS (**A**) and OS (**B**). Chemo, chemotherapy; CI, confidence interval; HR, hazard ratio; ICI, immune checkpoint inhibitor; ILD, interstitial lung disease; OS, overall survival; PD-L1, programmed death-ligand 1; PFS, progression free survival.

**Table 1:** Patients’ characteristics.

Characteristics	Total (N = 343)	≥75 Years (*n* = 132)	<75 Years (*n* = 211)	*p*-Value
Age (median, range)	73 (46–92)	79 (75–92)	69 (46–74)	
Sex				
Male	297 (87%)	114 (86%)	183 (87%)	1.0000
Female	46 (13%)	18 (14%)	28 (13%)	
Smoking history				
Current or former	335 (98%)	127 (96%)	208 (99%)	0.2686
Never	8 (2%)	5 (4%)	3 (1%)	
Histology				
Pure SCLC	323 (94%)	125 (95%)	198 (94%)	0.4574
Combined SCLC	21 (6%)	7 (5%)	14 (7%)	
Comorbidities				
With ILD	59 (17%)	32 (24%)	27 (13%)	0.008
ECOG PS				
0	35 (10%)	11 (8%)	24 (11%)	0.4812 *
1	194 (57%)	74 (56%)	120 (57%)	
2	66 (19%)	29 (22%)	37 (18%)	
3	46 (13%)	17 (13%)	29 (14%)	
4	2 (1%)	1 (1%)	1 (1%)	
Brain metastases	76 (22%)	22 (17%)	54 (26%)	0.0614
Liver metastases	132 (39%)	47 (36%)	85 (40%)	0.4254
Treatment regimens				
CBDCA + ETP + Atezolizumab	120 (35%)	43 (33%)	77 (37%)	<0.0001 **
Platinum + ETP + Durvalumab	98 (29%)	22 (17%)	76 (36%)	
Platinum + ETP	124 (36%)	67 (51%)	57 (27%)	
CDDP + CPT-11	1 (0%)	0 (0%)	1 (1%)	

SCLC; small cell lung cancer, ILD; interstitial lung disease, ECOG PS; eastern cooperative oncology group performance status, CBDCA; carboplatin, ETP; etoposide, CDDP; cisplatin, CPT-11; irinotecan. * Two-group comparison between patients with PS 0–1 and those with PS 2–4. ** Two-group comparison according to the presence or absence of concomitant PD-L1 inhibitor treatment.

**Table 2:** Univariate and multivariate analysis by Cox’s proportional hazard’s model in patients treated with ICI/chemo (OS).

		Univariate			Multivariate		
		HR	95% CI	*p*-Value	HR	95% CI	*p*-Value
Age	(≥75/<75)	1.064	0.7459–1.5178	0.7322			
Sex	(M/F)	1.2142	0.7424–1.9860	0.4393			
Smoking history	(Current/never)	1.1048	0.4087–2.9862	0.8443			
Histology	(Pure SCLC/ combined SCLC)	1.9439	0.6195–6.0996	0.2546			
PS	(0–1/2–4)	0.6311	0.4467–0.8916	0.009	0.6136	0.4340–0.8676	0.0057
Liver metastasis	(+/−)	1.9283	1.3960–2.6636	<0.0001	1.9656	1.4206–2.7197	<0.0001
Brain metastasis	(+/−)	0.8819	0.6149–1.2645	0.4942			
PD-L1 inhibitor	Atezolizumab/ Durvalumab	1.0998	0.7999–1.5120	0.5582			

ICI; immune checkpoint inhibitor, HR; hazard ratio, CI; confidential interval, M; male, F; female, SCLC; small cell lung cancer, PS; performance status.

**Table 3:** PD-L1 inhibitor + chemotherapy induced adverse events (grade 3 or higher).

Characteristics	Total (*n* = 218)	75 Years or Older (*n* = 65)	<75 Years (*n* = 153)	*p*-Value
IrAE *	16 (7%)	4 (6%)	12 (8%)	0.7823
Grade				
3	12 (6%)	3 (5%)	9 (6%)	1
4	2 (1%)	1 (2%)	1 (1%)	0.5084
5	2 (1%)	0 (0%)	2 (1%)	1
Types of irAE				
ILD	6 (3%)	1 (2%)	5 (3%)	0.6718
Enteritis	3 (1%)	0 (0%)	3 (2%)	0.5562
Type 1 diabetes	2 (1%)	1 (2%)	1 (1%)	0.5084
Psoriasis	1 (0%)	1 (2%)	0 (0%)	0.2982
Thrombocytopenia	1 (0%)	1 (2%)	0 (0%)	0.2982
Liver dysfunction	1 (0%)	0 (0%)	1 (1%)	1
Encephalitis	1 (0%)	0 (0%)	1 (1%)	1
Adrenal failure	1 (0%)	0 (0%)	1 (1%)	1
Non-irAE *	151 (69%)	48 (74%)	103 (67%)	0.4227
Grade				
3	71 (33%)	23 (35%)	48 (31%)	0.6359
4	100 (46%)	29 (45%)	71 (46%)	0.8822
5	5 (2%)	2 (3%)	3 (2%)	0.6359
Types of non-irAE				
Neutropenia	134 (61%)	40 (62%)	94 (61%)	1
Leukopenia	29 (13%)	5 (8%)	24 (16%)	0.1306
Febrile neutropenia	23 (11%)	5 (8%)	18 (12%)	0.4735
Thrombocytopenia	16 (7%)	7 (11%)	9 (6%)	0.2407
Anemia	7 (3%)	3 (5%)	4 (3%)	0.4281
Pneumonia	6 (3%)	1 (2%)	5 (4%)	0.6718
Hyponatremia	5 (2%)	2 (3%)	3 (2%)	0.6359
Sepsis	2 (1%)	0 (0%)	2 (1%)	1
Gastrointestinal bleeding	2 (1%)	0 (0%)	2 (1%)	1
Bradycardia	1 (0%)	1 (2%)	0 (0%)	0.2982
Liver disorder	1 (0%)	1 (2%)	0 (0%)	0.2982
Kidney disorder	1 (0%)	1 (2%)	0 (0%)	0.2982
Myocardial infarction	1 (0%)	1 (2%)	0 (0%)	0.2982
Loss of appetite	1 (0%)	1 (2%)	0 (0%)	0.2982
Hypokalemia	1 (0%)	1 (2%)	0 (0%)	0.2982
Pyelonephritis	1 (0%)	0 (0%)	1 (1%)	1
Drug hypersensitivity syndrome	1 (0%)	0 (0%)	1 (1%)	1
Catheter-related bloodstream infection	1 (0%)	0 (0%)	1 (1%)	1
Nausea	1 (0%)	0 (0%)	1 (1%)	1
Spasm	1 (0%)	0 (0%)	1 (1%)	1
Thrombosis	1 (0%)	0 (0%)	1 (1%)	1
Empyema	1 (0%)	0 (0%)	1 (1%)	1

IrAE; immune-related adverse events, ILD; interstitial lung disease. * The numbers for irAEs and non-irAEs represent the numbers of patients, whereas all other values represent the numbers of events for each type of AE.

## Data Availability

Dataset available on request from the authors.

## Supplementary Materials

The following supporting information can be downloaded at: https://www.mdpi.com/article/10.3390/cancers18193131/s1, Figure S1: Comparison of PFS and OS between patients aged ≥75 and <75 years treated with PD-L1 inhibitor plus chemotherapy, Figure S2: PFS and OS in patients aged 75 years or older, including those with ILD, Figure S3: Comparison of PFS between ICI plus chemotherapy and chemotherapy alone in patients aged ≥75 years with good or poor PS, Table S1: Distribution of Treatment Modalities by Year of Treatment Initiation, Table S2: Reasons for not combining PD-L1 inhibitors, Table S3: Patients’ characteristics (75 years or older), Table S4: Univariate and multivariate analysis by Cox’s proportional hazard’s model in elderly patients group (OS), Table S5: Adverse events in elderly patients (grade 3 or higher), Table S6: Subsequent therapies following 1st-line treatment.
