# Supplementary material for: A Retrospective Study of the Efficacy and Safety of Chemoimmunotherapy in Elderly Patients with Extensive-Stage Small Cell Lung Cancer
Source: Cancers (Basel). 2026 Sep 27;18(19):3131. doi: 10.3390/cancers18193131 (PMC13664149; doi:10.3390/cancers18193131)
Supplement: Supplementary file 1 [file cancers-18-03131-s001.zip › Figure S2 revised.pdf]

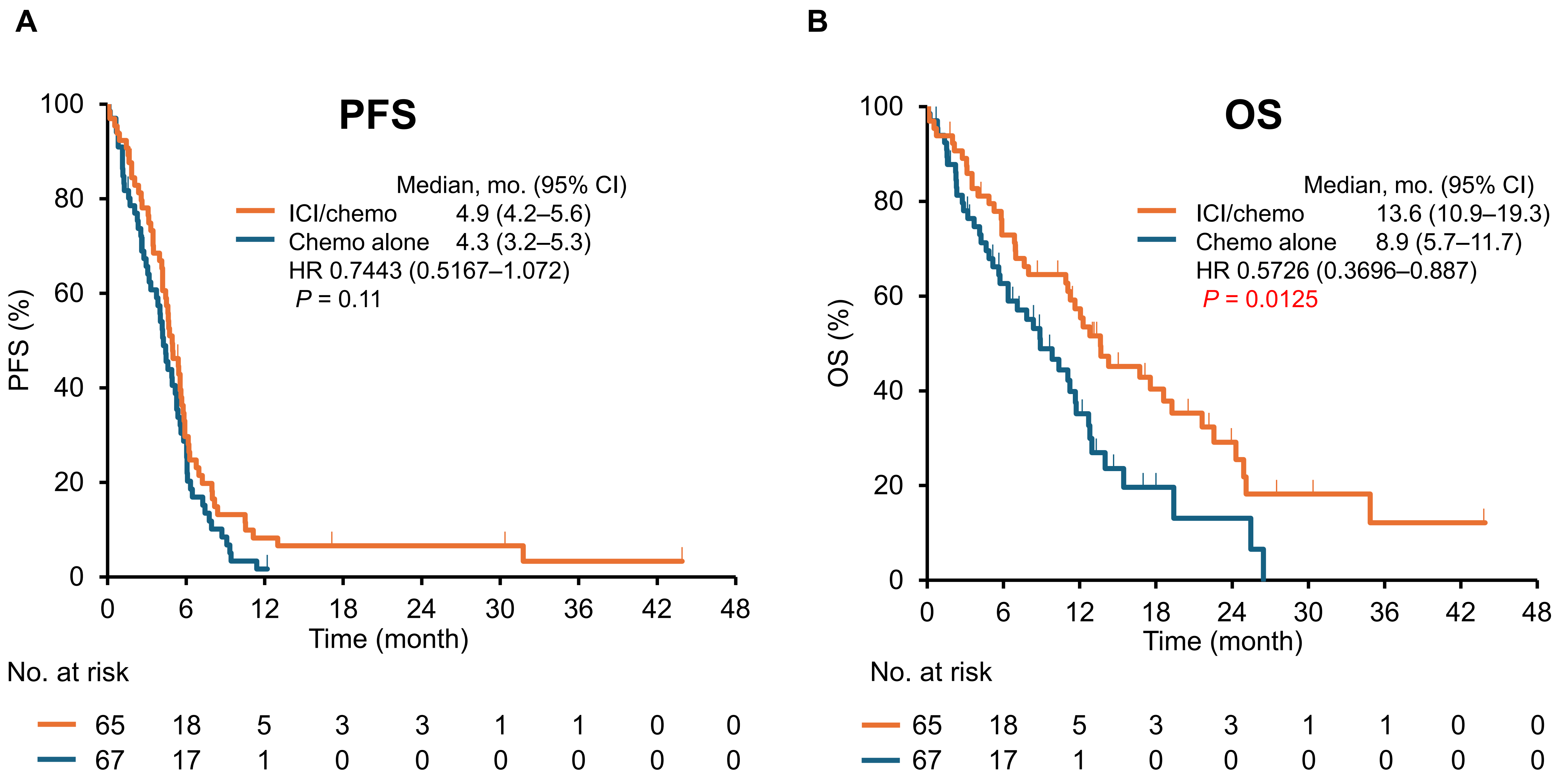

Figure S2. PFS and OS in patients aged 75 years or older, including those with ILD.
(A) PFS and (B) OS in patients aged ≥75 years treated with PD-L1 inhibitor + chemotherapy or chemotherapy alone.
ICI; immune checkpoint inhibitor, PFS; progression free survival, OS; overall survival, HR; hazard ratio,
CI; confidence interval, Chemo; chemotherapy, ILD; interstitial lung disease.
