# Supplementary material for: A Retrospective Study of the Efficacy and Safety of Chemoimmunotherapy in Elderly Patients with Extensive-Stage Small Cell Lung Cancer
Source: Cancers (Basel). 2026 Sep 27;18(19):3131. doi: 10.3390/cancers18193131 (PMC13664149; doi:10.3390/cancers18193131)
Supplement: Supplementary file 1 [file cancers-18-03131-s001.zip › Figure S3 revised.pdf]

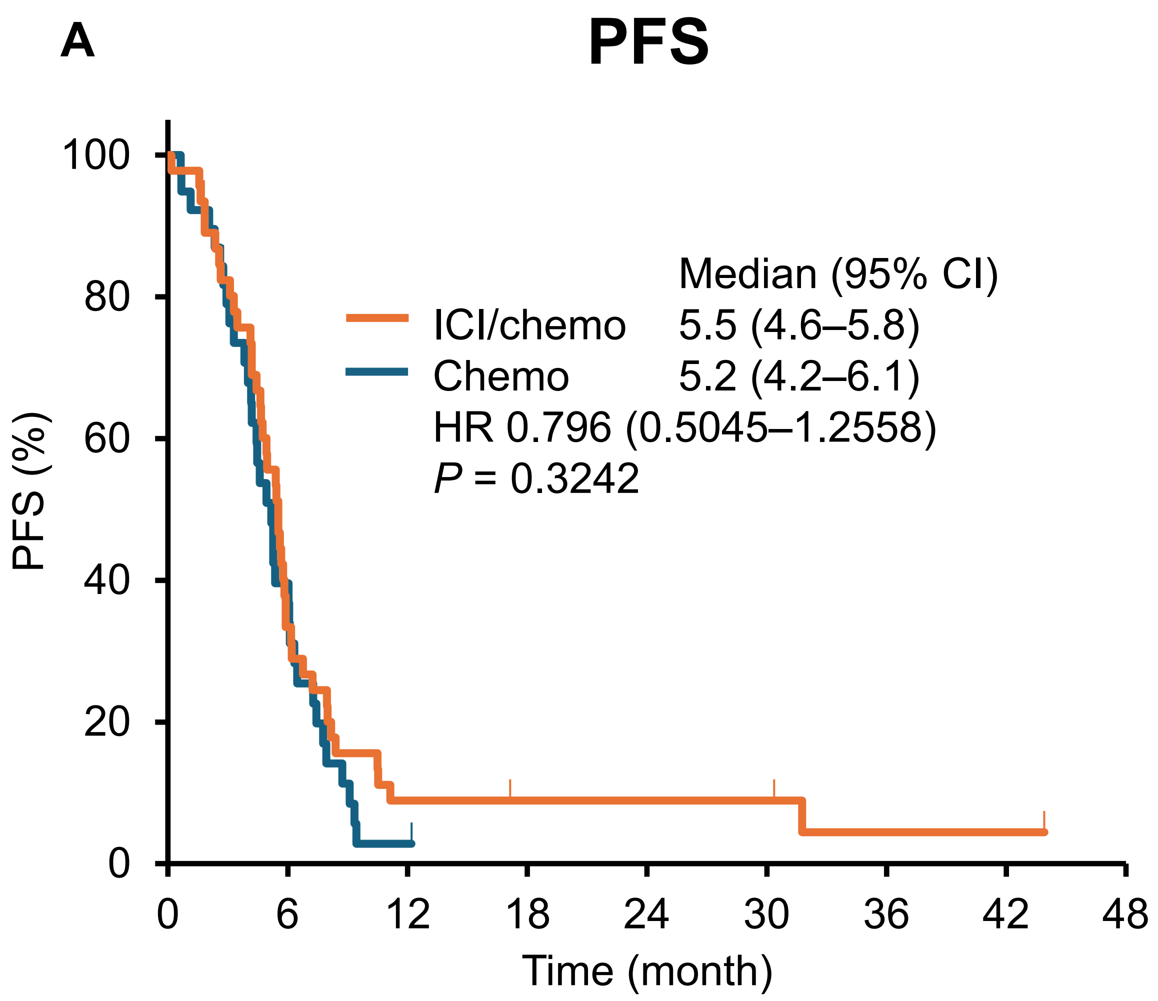

No.at risk

ICI/Chemo	46	15	4	3	3	3	1	1	0
Chemo	39	21	1	0	0	0	0	0	0

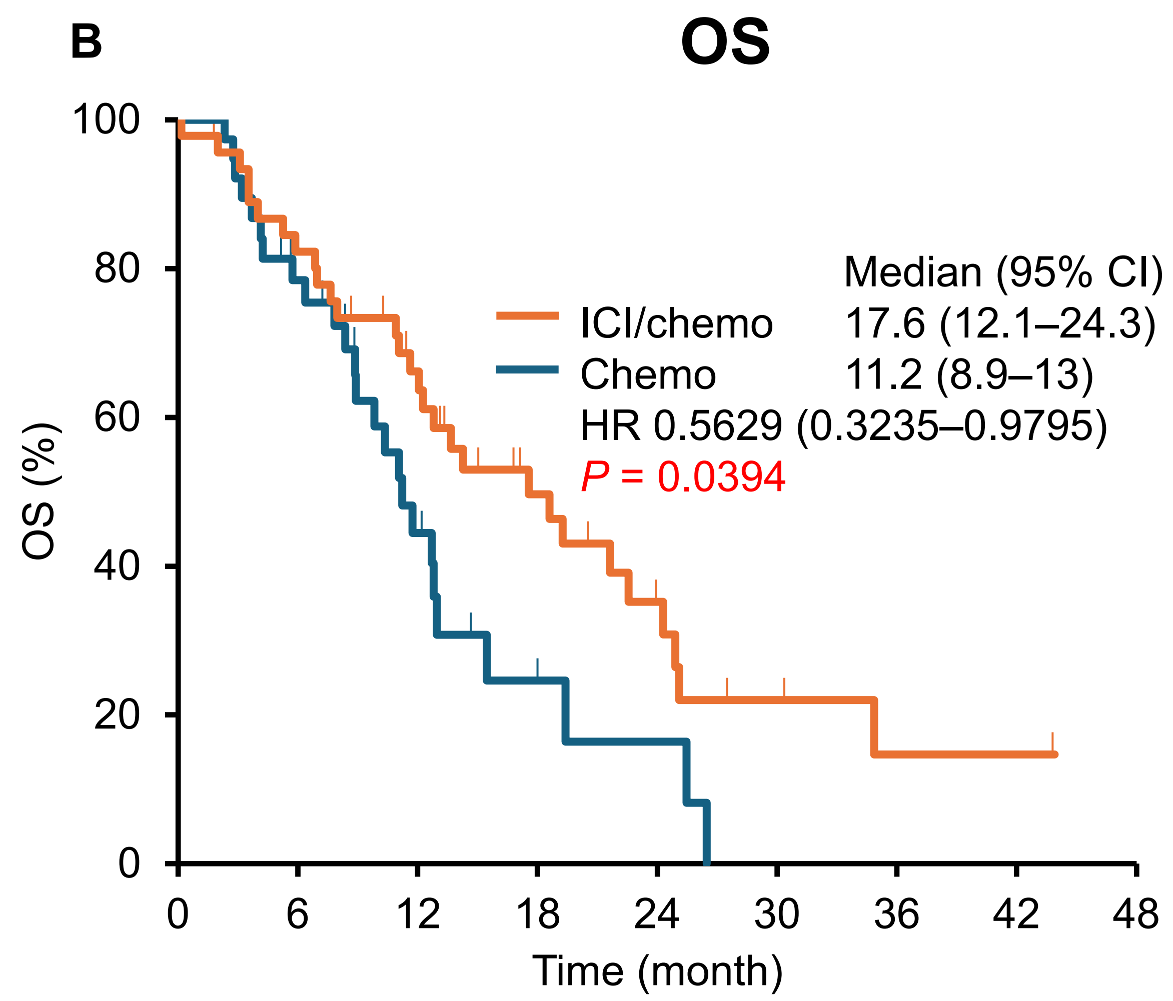

No.at risk

ICI/Chemo	46	37	26	15	8	4	2	2	0
Chemo	39	26	12	4	2	0	0	0	0

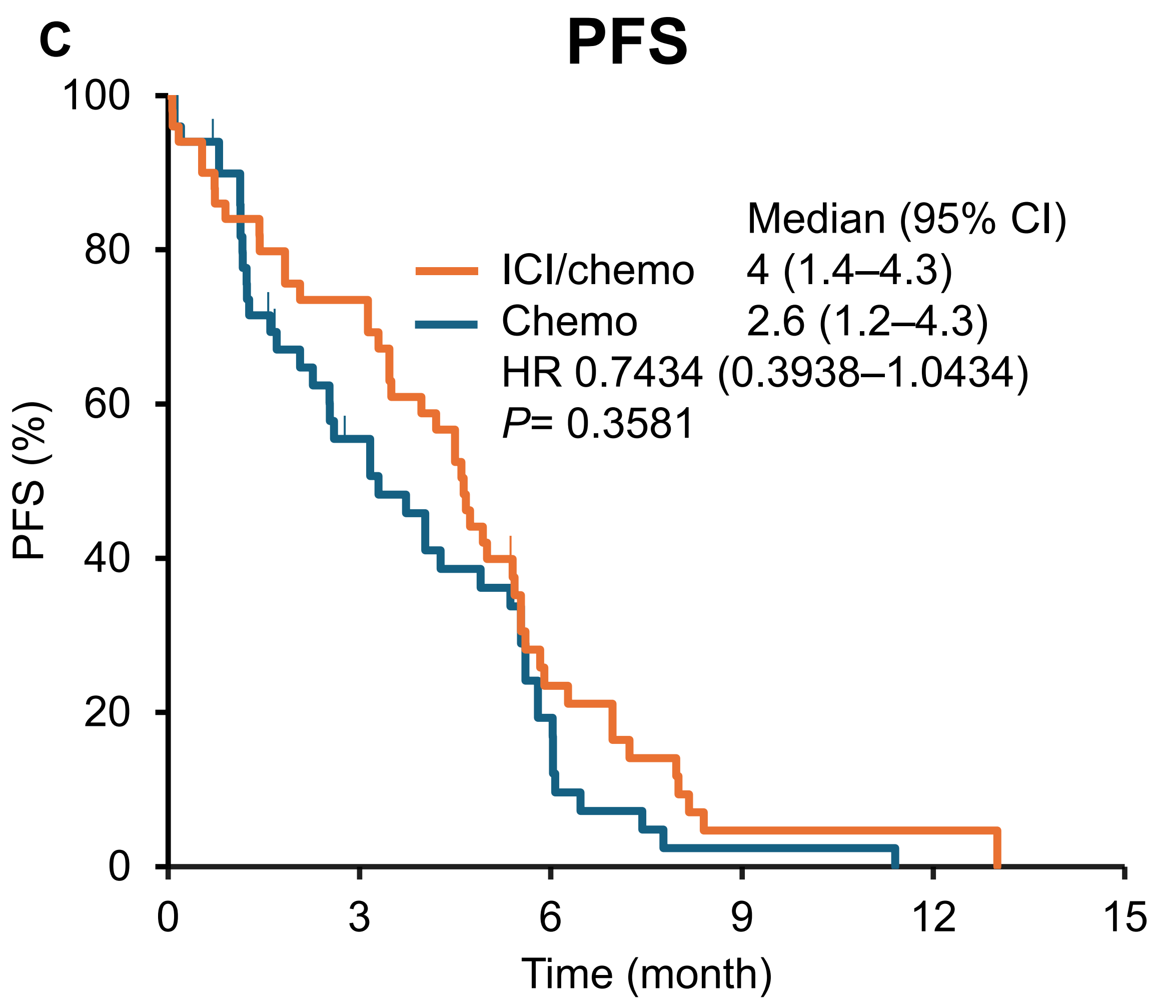

No.at risk

ICI/Chemo	19	12	3	2	1	0
Chemo	28	11	3	1	0	0

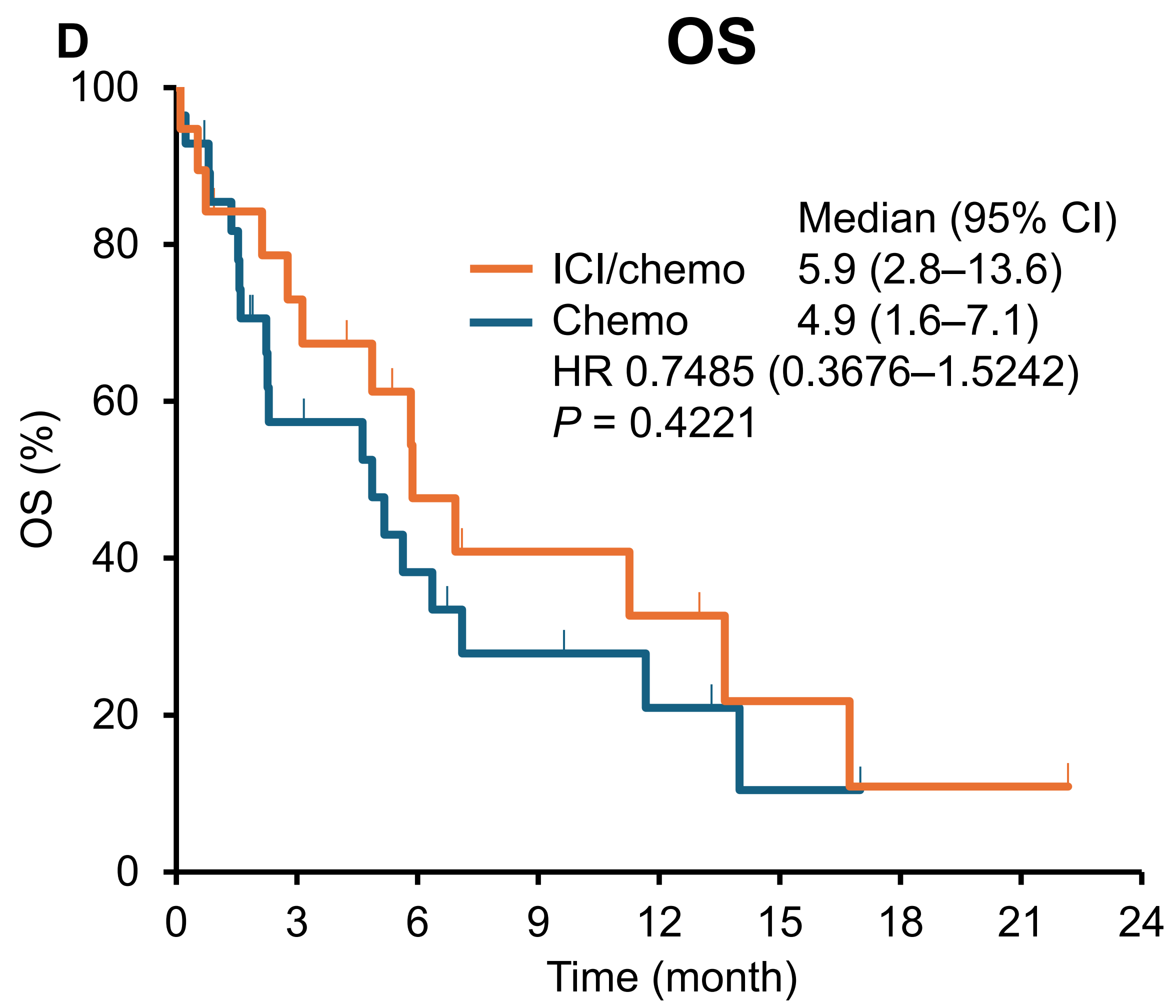

No.at risk

ICI/Chemo	19	13	7	5	4	2	2	2	0
Chemo	28	13	8	5	3	1	0	0	0

Figure S3. Comparison of PFS between ICI plus chemotherapy and chemotherapy alone in patients aged ≥75 years with good or poor PS

(A) PFS and (B) OS in patients treated with PD-L1 inhibitor plus chemotherapy and chemotherapy with good PS (PS 0-1). (C) PFS and (D) OS in patients treated with PD-L1 inhibitor plus chemotherapy and chemotherapy with poor PS (PS 2-4). ICI; immune checkpoint inhibitor, PS; performance status, PFS; progression free survival, OS; overall survival, HR; hazard ratio, CI; confidence interval, Chemo; chemotherapy.
